# A Virtual Home for the Virtual Clinical Trial

**DOI:** 10.2196/15582

**Published:** 2020-01-03

**Authors:** Susan Persky

**Affiliations:** 1 Social and Behavioral Research Branch National Human Genome Research Institute Bethesda, MD United States

**Keywords:** virtual reality, virtual clinical trials, eHealth

## Abstract

Virtual clinical trials (VCTs) can satisfy the need for rigorous clinical trials by using distributed technological solutions that eliminate the need for a physical trial site. This report explores potential benefits of using virtual reality (VR) to provide a “virtual site” for VCTs, a shared immersive hub in which VCT participants could experience elements of the trial and interact with the trial team. VR is a communication technology that has been emerging alongside the development of VCTs, although they have never been merged in a substantial way. Many of the gaps within the VCT paradigm are areas in which VR excels. VR environments are standardized and precisely uniform, the technology allows introduction of an almost endless set of stimuli to participants’ visual and auditory systems, and VR systems are adept at capturing precise movement and behavioral data. Although VR has not yet found its way into VCTs, much of the groundwork for such integration has been laid through research and technological development achieved in the past few years. Future implementation of VR within VCTs could move us from site-less trials to those with a virtual site serving as a hub for trial information provision, interaction with trial representatives, administration of evaluations and assessments, and more.

## Introduction

Rapid innovation in health and health care technologies, interventions, and products require the attendant proliferation of rigorous clinical trials to evaluate their efficacy and effectiveness. To meet these needs, the field has been slowly moving toward increasing adoption of virtual clinical trials (VCTs). VCTs go by many names (eg, site-less, remote, decentralized, direct-to-patient, and patient-centered trials), but these generally involve evaluating the effect of a clinical intervention (often a pharmaceutical product) within research participants’ own settings, as opposed to a clinical trial site. They satisfy the need for rigorous clinical trials performed among a diverse sampling of the appropriate population, using benefits conferred by distributed technological solutions (eg, mobile phone apps) that are often already present in the homes and routines of research participants under study. Boosters of the model submit that VCTs can reduce costs, shorten trial timelines, increase protocol adherence, and boost recruitment numbers and participant diversity, while simultaneously allowing for continuous real-world data collection in the context of real-life settings and events [1,2]. These trials typically provide participant access to research teams through Web-based portals, sometimes provide home visits, and collect data through networked wearables and medical devices, surveys, and other means. Although there are certainly complexities related to the massive amount of data generated, safety concerns, and other considerations, this approach holds great potential for growth. Indeed, if we think beyond trials involving pharmaceuticals and therapeutics, these site-less trials have been flourishing for years in the context of evaluating products and interventions (such as mobile health apps) intended to be widely deployed in free-living environments.

**Figure 1:**
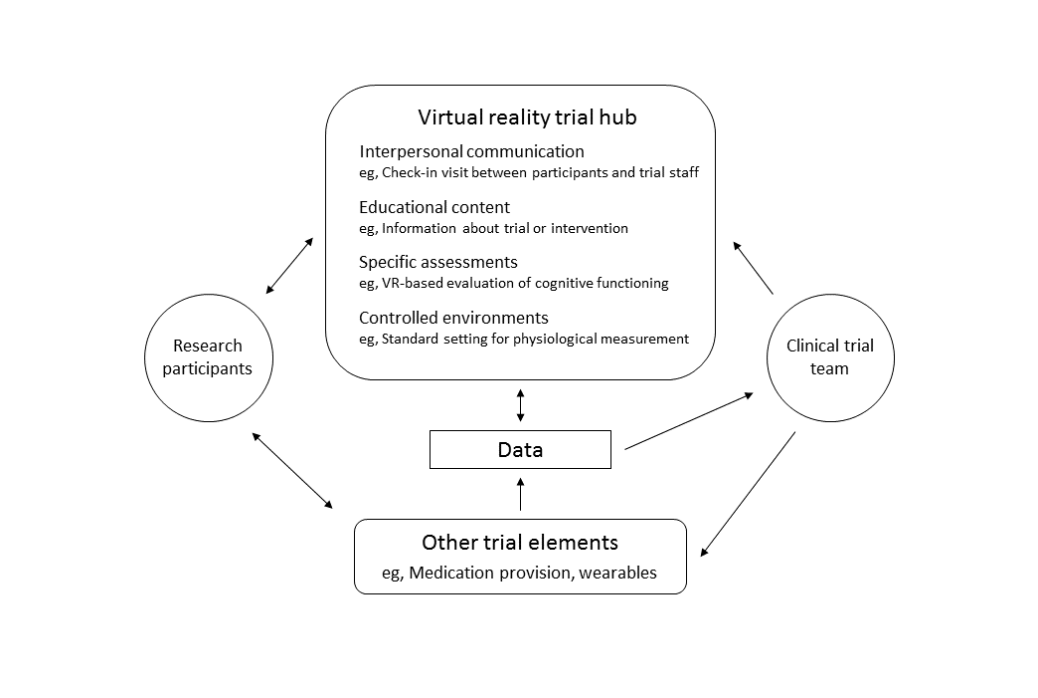
Relationships among virtual clinical trial elements incorporating virtual reality.

Still, since the dawn of the VCT, there has been argument for retaining the clinical site. Distributing data collection introduces a lack of standardization, a lack of relationship between the trial team and participants, the inability to collect certain types of data, and a lack of researcher knowledge about the contextual details of data collection, including whether the intended individual is participating in trial activities [3]. For these and other reasons, there may be benefits to providing a “virtual site” for VCTs—a shared immersive hub in which every VCT participant could experience trial elements and interact with the trial team (Figure 1).

## Virtual Reality to Augment Virtual Clinical Trials

It is time to consider whether and how virtual reality (VR) could be used to augment VCTs. VR is a communication technology that has been emerging alongside VCTs, and although they share a component of their name, they have not been merged in a substantial way. There are several arguments for doing so. Many of the gaps left by the VCT paradigm are areas in which VR excels. VR environments are digital; thus, they are very precisely uniform, and the technology allows introduction of an almost endless set of stimuli to participants’ visual and auditory systems (and sometimes other sensory systems as well). VR is adept at capturing precise movement and behavior data; indeed, user movement drives the very operation of VR systems. Such data collection could also provide information on participant adherence to elements of the trial regimen, as well as data points to demonstrate that the intended individual is participating in the remote trial. Indeed, individuals’ VR data are unique and identifiable in terms of elements such as eye height, gait, and movement patterns [4]. Although other related technologies, such as mobile phone apps, and nonimmersive virtual environments may likewise confer benefits for VCTs, these particular elements (immersion in uniform but realistic simulations, unique data signatures generated by body movement) are unique to VR.

## Virtual Reality Research Relevant for Remote Interactions

With the aging of the American population, evaluation and therapy in home settings have become a major focus within the VR industry. For example, researchers have begun to assess the ability of patients to use VR apps for physical and occupational therapy self-assessment and exercises in the home, with generally favorable results [5-7]. This research has sensibly occurred primarily in domains where mobility can be an issue and where home-based activities are already the norm. This is a potential starting point for VR-based motion or mobility assessments in VCTs where interventions under study influence these processes. This is, however, only a single area of development. Given a very active VR development community around health, health care, and wellness, there are many use cases soon to be ripe for the picking. For example, VR assessments of neuropsychological processes and outcomes have been developed both in the context of noninterventional natural history trials and in pharmaceutical trial contexts. For example, in laboratory-based work, researchers have used VR classrooms and driving simulators to evaluate effects of psychostimulants [8-10]. Researchers have also developed VR environments to elicit and assess stress reactivity in both laboratory-standard and in ecologically realistic ways [11-13]. Cognitive and executive function evaluation is another area of active VR development, wherein testing takes place in a lifelike virtual environment (eg, a virtual grocery store) to provide consistency between trials and between patients [14]. Elicitation and evaluation of craving and substance use behavior using VR environments have become quite sophisticated in recent years [15,16], alongside VR-based food choice measures [17-19]. These tools could provide standardized environments in which to measure specific reactions and behaviors associated with interventions under study. They also allow evaluation in the face of such stimuli without sending participants into the way of potential harm. Although many VR environments are typically designed to bring elements of the real world (bars, liquor stores, cafeterias, etc) into sterile laboratory environments, they could provide standardized environments within a home context just as compellingly.

In addition, note that applications of VR to VCTs need not be complicated or complex. Consider measuring resting blood pressure among VCT participants in the variety of home environments where this might occur—alone in a quiet room, versus surrounded by active children, watching a cooking show versus watching a true-crime show. Now consider what might be gained by having these measurements taken while all participants are relaxing on the same VR beach watching the waves roll in.

## Interpersonal Factors in Virtual Clinical Trials

Also critical is the social component of clinical trials, along with the trust that can develop between participants and researchers. A VR-based communication hub for VCTs could reinsert some of the human element into these distributed studies. Many researchers have created VR clinical settings that could be leveraged as familiar and trustworthy contexts in which to convey clinical information to patients. Research on social VR, as well as work on telemedicine, has shown the ability of VR to support true social interactions and therapeutic alliance [20,21]. Use of VR to support interpersonal interaction between patients and trial staff could also be beneficial in the case of single-blind trials, wherein communication could be filtered or elements could be automated to reduce concerns about researcher expectations seeping into the encounter [22]. Although VR can convincingly mimic a clinical interaction [23,24], there may also be reason to embellish and explore new possibilities in these information exchanges. Patient education could be enhanced by bringing VCT participants into VR educational environments, for example, to help patients visualize health and medical data [25,26]. Clinical trialists could have a full arsenal of VR visualizations and demonstrations when explaining trial procedure, disease processes, medical procedures, treatment methods, and so on, within the consent process and as trials proceed.

## Why Now?

Deploying VR to participant home environments has become increasingly possible because of massive growth in VR technology in recent years, as well as (much more slowly) growing ownership of VR hardware among consumers. VR hardware is available in a variety of form factors and price points. Although eventually, many trial participants may be able to bring their own VR equipment to trials, much as they might bring their own computer or mobile phone, at present, this would be an unlikely scenario. However, trial-provided equipment is common, and certainly, increased control over and standardization of trial equipment are likely of benefit at this stage.

## Limitations

This is not to suggest that VR could or should be integrated into all, or even most, VCTs. There will be a great variation in the potential benefits of VR for a given trial, and these should be always weighed against potential risks and downsides. Already identified risks of VR in research include privacy and data security, as well as potential health and comfort risks of equipment use [27]. There are also some VR tools that, although effective, are best experienced while under the direct care of a health care professional, such as those aimed at addressing posttraumatic stress disorder [28]. These applications may not be appropriate for remote use. There may additionally be populations for whom home VR use will not be appropriate (eg, individuals with certain neurological conditions). Finally, as with all VCT tools, care must be taken to ensure that use protocols are easy to understand, seamless, and free of frustrations for participant populations.

## Conclusions

VCTs and VR have grown up alongside but parallel to one another. The next steps toward enabling the integration of VR within VCTs include increasing distributed, site-less research on VR-based interventions. Indeed, clinical research within the VR research community itself is still somewhat emerging [29,30]. Most VR-centric health and medical research is currently performed in laboratory or medical settings; few evaluation trials have been distributed into individuals’ home contexts. By taking several smaller steps, such conducting more VCTs evaluating VR-based interventions and using VR tools as part of traditional clinical trials, the frameworks and optimal approaches for engaging research participants and integrating VR into workflows can be developed. As such, we can outline the pathway moving from site-less VCTs to trials that have a virtual site, serving as a hub for trial information provision, interaction with the trial team, administration of assessments, and more.

## Abbreviations

VCT: virtual clinical trials
VR: virtual reality
